# Double Trouble: Renal Collision Tumors with Unexpected Histology—A Case Report with Review of Literature

**DOI:** 10.15586/jkc.v13i3.479

**Published:** 2026-08-15

**Authors:** Puneeth Medapati, Harshdeep Singh, Thiyagarajan Kalaiselvi Aravind, Siddharth Yadav

**Affiliations:** 1 Department of Urology and Renal Transplant, VMMC & Safdarjung Hospital, Delhi, India;; 2 Department of Urology, Government Medical College, Amritsar, Punjab, India

**Keywords:** kidney, lymphoma, non-Hodgkin, RCC

## Abstract

Primary Non-Hodgkin’s Lymphoma (NHL) involving the adrenal gland is rare, and its synchronous occurrence with renal cell carcinoma (RCC) is exceedingly uncommon. Although adrenal involvement is observed in approximately 20% of systemic NHL cases, primary adrenal lymphoma accounts for only a small proportion of these cases. RCC accounts for 2–3% of all malignancies and is the second most common genitourinary cancer. The coexistence of RCC with other primary malignancies has been described, often in the context of familial syndromes; however, its synchronous presentation with primary adrenal NHL is exceptionally rare. A 54-year-old male with diabetes presented with intermittent dull left flank pain for 18 months and a recent loss of appetite. Contrast-enhanced computed tomography revealed a large, heterogeneous, hyper-enhancing mass in the left suprarenal region replacing the adrenal gland, along with a separate enhancing exophytic lesion in the left kidney. Positron emission tomography showed high metabolic activity in both lesions and regional lymph nodes. The patient underwent open radical nephrectomy with adrenalectomy and regional lymph node dissection. Histopathology demonstrated diffuse large B-cell lymphoma involving the adrenal gland with extension into the kidney, perinephric tissue, and perihilar fat, along with a separate Grade II clear cell RCC in the lower pole of the kidney. Immunohistochemistry confirmed B-cell lineage lymphoma. Lymph nodes were negative for metastasis. The patient received nine cycles of CHOP (Cyclophosphamide, Doxorubicin [Hydroxydaunorubicin], Vincristine [Oncovin], and Prednisolone) chemotherapy and remains recurrence-free at the 6-month follow-up. This case highlights a rare synchronous occurrence of primary adrenal NHL and ipsilateral clear cell RCC. Shared genetic or immunological mechanisms may underlie this association, warranting further investigation.

## Introduction

Primary non-Hodgkin’s Lymphoma (NHL) of the adrenal gland is a rare extranodal malignancy, despite adrenal involvement being reported in up to 20% of patients with disseminated lymphoma. Primary adrenal lymphoma accounts for only a small fraction of all NHL cases and is most commonly of the diffuse large B-cell subtype (1). Renal cell carcinoma (RCC), on the other hand, accounts for approximately 2–3% of all adult malignancies and is the second most common genitourinary cancer. Clear cell RCC (ccRCC) is the predominant histological subtype. Although RCC has been described in association with other primary malignancies, particularly in the setting of hereditary cancer syndromes, its synchronous occurrence with primary adrenal NHL is exceedingly uncommon. The coexistence of these two distinct malignancies within the same organ system raises important diagnostic and therapeutic considerations (2). We report a rare case of synchronous ipsilateral primary adrenal NHL and ccRCC and discuss their possible pathogenetic associations.

## Case Presentation

A 54-year-old male presented with intermittent dull aching pain in the left flank for 1.5 years. The pain was insidious in onset, non-radiating, and was not associated with hematuria, fever, or lower urinary tract symptoms. Over the preceding 3 weeks, he reported loss of appetite and generalized weakness. There was no history of weight loss, night sweats, or prior malignancy. He had a 4-year history of diabetes mellitus managed with oral hypoglycemic agents and had no history of smoking or a significant family history of cancer.

Physical examination was unremarkable, with no palpable abdominal mass or lymphadenopathy. Laboratory investigations, including hemogram, renal function tests, and liver function tests, were within normal limits.

**Figure 1:**
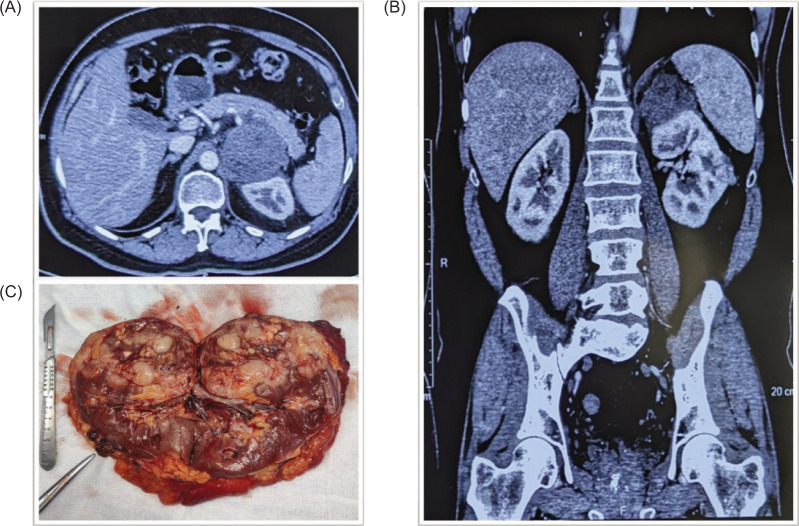
Cross-sectional imaging and resected specimen. (A) Axial contrast-enhanced CT image showing a right upper pole renal mass with associated adrenal lesion. (B) Coronal contrast-enhanced CT image demonstrating the renal and adrenal tumors in relation to surrounding structures. (C) Cut section of the resected specimen displaying the renal mass on gross examination.

Contrast-enhanced computed tomography (CECT) revealed an 8.2 × 7.2 × 7 cm heterogeneous, hyper-enhancing lesion in the left suprarenal region with irregular margins and non-visualization of the left adrenal gland, suggestive of adrenal replacement (Figure 1A). A separate 2.6 × 2.7 × 2.1 cm hyper-enhancing exophytic lesion was noted along the lateral aspect of the left kidney (Figure 1B). Positron emission tomography–computed tomography (PET-CT) demonstrated a metabolically active 6.6 × 6.9 × 7 cm left suprarenal mass (maximum standardized uptake value [SUVmax] 11.36) and a 2.3 × 2.2 cm hypermetabolic lesion in the left kidney (SUVmax 4.77). Radiotracer uptake was also noted in the perilesional, gastrosplenic, and gastrohepatic lymph nodes.

**Figure 2:**
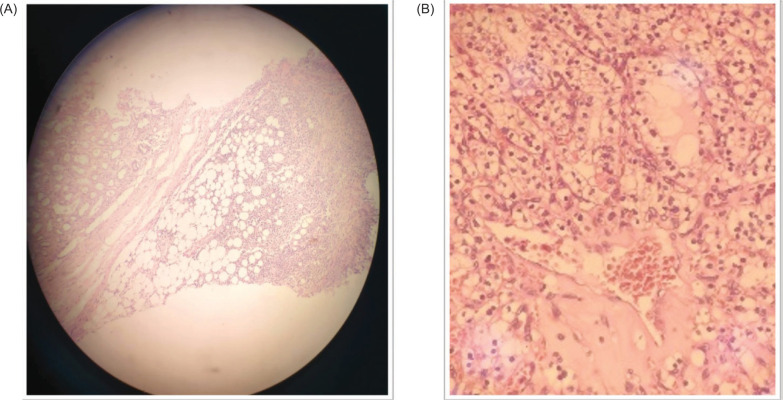
Tumor histopathology (hematoxylin & eosin stain). (A) Photomicrograph showing features consistent with clear cell renal cell carcinoma (ccRCC), with tumor cells arranged in nests and alveolar patterns, separated from adjacent uninvolved renal parenchyma. The cells exhibit clear cytoplasm and distinct cell borders (10× magnification, H&E). (B) Photomicrograph demonstrating diffuse infiltration by atypical lymphoid cells consistent with Non-Hodgkin lymphoma (NHL), showing sheets of monotonous lymphoid cells with scant cytoplasm and hyperchromatic nuclei (10× magnification, H&E).

The patient underwent open radical nephrectomy with adrenalectomy and regional lymph node dissection. Intraoperatively, a large suprarenal mass replacing the adrenal gland, along with enlarged pre- and para-aortic lymph nodes, was observed. Gross examination revealed a grey-white lobulated tumor with areas of necrosis and a thin rim of residual adrenal tissue, with invasion of the upper pole of the kidney (Figure 1C). Microscopy revealed diffuse sheets of pleomorphic round-to-oval cells with prominent nucleoli and brisk mitotic activity. A separate well-circumscribed tumor in the lower pole demonstrated polygonal cells with clear cytoplasm, consistent with Grade II clear cell RCC (Figures 2A and 2B). Immunohistochemistry (IHC) confirmed B-cell NHL. Lymph nodes were negative for metastasis. Chemotherapy was initiated 2 weeks after the histopathological diagnosis. Following nine cycles of CHOP (Cyclophosphamide, Doxorubicin [Hydroxydaunorubicin], Vincristine [Oncovin], and Prednisolone) chemotherapy, the patient remained free of disease recurrence during the 6-month follow-up period.

## Discussion

The synchronous occurrence of NHL and RCC is uncommon, and the coexistence of primary adrenal NHL with ipsilateral ccRCC is exceptionally rare. Although secondary adrenal involvement is relatively frequent in disseminated lymphoma, primary adrenal lymphoma accounts for less than 1% of all extranodal lymphomas (3). The adrenal glands lack native lymphoid tissue, making primary lymphomatous involvement biologically intriguing. Most reported cases are diffuse large B-cell lymphomas and often present with bilateral adrenal masses, adrenal insufficiency, or systemic “B” symptoms. In contrast, our patient presented with unilateral adrenal involvement without adrenal insufficiency, making the diagnosis more challenging.

Renal cell carcinoma is well known for its association with second primary malignancies. Epidemiological evidence supports a bidirectional relationship between NHL and RCC. Data derived from the Surveillance, Epidemiology, and End Results (SEER) database have demonstrated a significantly higher-than-expected incidence of RCC among patients with NHL and vice versa. Importantly, many of these associations occur metachronously and are often attributed to the long-term effects of chemotherapy, radiotherapy, or immune dysregulation. However, synchronous presentation, particularly before systemic therapy, suggests alternative pathogenetic mechanisms beyond treatment-related carcinogenesis. Chen et al., analyzing SEER data from 1973 to 2006, identified 181,009 patients with NHL and 118,122 patients with RCC. Among these, 1039 patients had both malignancies, a number significantly higher than the expected 24 cases (4).

Several hypotheses have been proposed to explain this association. One major consideration is immune dysregulation. NHL itself reflects abnormal lymphoid proliferation resulting from genetic and immunological alterations. Impaired immune surveillance may predispose patients to additional primary malignancies, including RCC. Conversely, RCC is recognized as an immunogenic tumor, with tumor-infiltrating lymphocytes and responsiveness to immunotherapy underscoring its interaction with the immune system. Dysregulated cytokine networks, chronic antigenic stimulation, or shared immune escape pathways may contribute to the simultaneous development of both malignancies. Case reports in the literature further support this rare association. Ewa et al. described a 74-year-old woman with large B-cell lymphoma of the neck with bilateral RCC and oncocytoma (5). Nishikubo et al. reported a series of eight cases of RCC associated with lymphoid malignancies; however, these tumors did not occur synchronously (6).

Genetic susceptibility represents another plausible mechanism. Clear cell RCC is strongly associated with alterations in the Von Hippel–Lindau (VHL) tumor suppressor gene located on chromosome 3p (7). Loss of VHL function leads to the stabilization of hypoxia-inducible factors (HIFs) and the upregulation of angiogenic mediators such as vascular endothelial growth factor (VEGF). Although primarily implicated in RCC, abnormalities involving chromosome 3p have also been described in certain lymphoid malignancies. Similarly, deletions of chromosome 17p and mutations in the p53 tumor suppressor gene have been identified in both RCC and NHL (8, 9). While these shared cytogenetic abnormalities do not establish causation, they suggest overlapping molecular pathways that may predispose susceptible individuals to dual malignancies.

Cytokine dysregulation is another area of interest. Interleukin-6 (IL-6), a proinflammatory cytokine, has been implicated in the pathogenesis of both RCC and NHL. Elevated IL-6 levels can promote tumor proliferation, angiogenesis, and resistance to apoptosis (10). Similarly, VEGF overexpression has been demonstrated in both tumors, facilitating angiogenesis and tumor growth. Cluster of differentiation 44 (CD44), a cell surface adhesion molecule involved in tumor invasion and metastasis, has also been reported to be elevated in both RCC and certain lymphomas (11). These molecular parallels suggest that shared signaling pathways could potentially underlie synchronous tumor development.

From a diagnostic standpoint, distinguishing between adrenal metastasis from RCC and primary adrenal lymphoma is critical. RCC frequently metastasizes to the adrenal gland, particularly on the ipsilateral side (12). Radiologically, both entities may present as adrenal masses with heterogeneous enhancement. However, high metabolic activity on PET-CT, particularly with markedly elevated SUVmax, may raise suspicion for lymphoma (13). Definitive diagnosis relies on histopathological examination and IHC. In our case, the adrenal tumor demonstrated diffuse sheets of pleomorphic lymphoid cells positive for leukocyte common antigen (LCA), CD19, CD20, and B-cell lymphoma 2 (BCL-2), confirming B-cell lineage lymphoma. The renal lesion displayed classic morphological features of clear cell RCC, with polygonal cells and clear cytoplasm arranged in nests separated by delicate fibrovascular septa.

Therapeutically, the management of synchronous malignancies poses challenges. Surgical resection is the standard of care for localized RCC and also provides diagnostic clarity. In contrast, primary adrenal lymphoma is primarily treated with systemic chemotherapy. In the present case, radical nephrectomy with adrenalectomy allowed complete excision of both tumors and an accurate histopathological diagnosis. The absence of lymph node metastasis favored localized disease. Rituximab-CHOP administered for six cycles is considered the standard first-line therapy for primary adrenal diffuse large B-cell lymphoma. However, in the present case, rituximab could not be administered because of financial constraints, and an extended course of nine cycles of CHOP chemotherapy was given based on the treating oncologist’s judgment (14). The favorable response and absence of recurrence during follow-up highlight the importance of multimodal management.

Prognosis in primary adrenal lymphoma is generally considered poor due to its aggressive behavior and frequent bilateral involvement. Factors associated with worse outcomes include advanced age, bilateral adrenal disease, elevated lactate dehydrogenase, and adrenal insufficiency. However, early diagnosis and prompt chemotherapy can significantly improve survival (15). In contrast, prognosis in RCC depends on tumor stage, grade, and histological subtype. The small size and Grade II histology of the RCC in our patient suggest a favorable prognosis for the renal cancer.

This case underscores the need for thorough evaluation of adrenal masses detected in patients with renal tumors. Not all adrenal lesions in the setting of RCC represent metastatic disease. Failure to recognize a synchronous primary lymphoma could delay appropriate systemic therapy and adversely affect outcomes. Multidisciplinary collaboration involving urologists, oncologists, radiologists, and pathologists is essential in managing such complex presentations.

## Conclusion

Synchronous primary adrenal NHL and ipsilateral ccRCC represent an exceedingly rare clinical entity. While epidemiological and molecular evidence suggests possible shared pathogenic mechanisms, the exact relationship between these malignancies remains unclear. Immune dysregulation, genetic susceptibility, and cytokine-mediated tumorigenesis may play contributory roles. Awareness of this rare association is crucial for accurate diagnosis and optimal management. Further studies and the accumulation of similar cases are required to better understand the biological interplay between these malignancies and to determine whether their coexistence represents a coincidence or a shared oncogenic pathway.

## Mandatory Disclosure on Use of Artificial Intelligence

The authors declare that no AI-assisted tools were used in the preparation of this manuscript. All references have been manually verified for accuracy and relevance.
